# Genetic Predisposition to Familial Nonmedullary Thyroid Cancer: An Update of Molecular Findings and State-of-the-Art Studies

**DOI:** 10.1155/2010/385206

**Published:** 2010-06-10

**Authors:** Elena Bonora, Giovanni Tallini, Giovanni Romeo

**Affiliations:** ^1^Unit of Medical Genetics, S.Orsola-Malpighi Hospital, 40138 Bologna, Italy; ^2^Dipartimento di Anatomia Patologica, Bellaria Hospital, University of Bologna, 40138 Bologna, Italy

## Abstract

Familial thyroid cancer has become a well-recognized entity in patients with thyroid cancer originating from follicular cells, that is, nonmedullary thyroid carcinoma. The diagnosis of familial thyroid cancer provides an opportunity for early detection and possible prevention in family members. Understanding the syndromes associated with familial thyroid cancer allows clinicians to evaluate and treat patients for coexisting pathologic conditions. About five percents of patients with well-differentiated thyroid carcinoma have a familial disease. Patients with familial non-medullalry thyroid cancer have more aggressive tumors with increased rates of extrathyroid extension, lymph node metastases, and frequently show the phenomenon of “anticipation” (earlier age at disease onset and increased severity in successive generations). So far, four predisposition loci have been identified in relatively rare extended pedigrees, and association studies have identified multiple predisposing variants for differentiated thyroid cancer. This suggests that there is a high degree of genetic heterogeneity and that the development of this type of tumor is a multifactorial and complex process in which predisposing genetic variants interact with a number of incompletely understood environmental risk factors. Thus, the search for the causative variants is still open and will surely benefit from the new technological approaches that have been developed in recent years.

## 1. Thyroid Cancer: Epidemiology and Clinicopathologic Features

Thyroid cancer is divided into two types, based on the cells of origin: differentiated thyroid cancer of follicular cell origin (NMTC) and medullary thyroid cancer (MTC) originating from parafollicular cells [1]. The molecular mechanisms underlying MTC development, mainly due to gain-of-function mutations of the proto-oncogene RET have been studied in depth and are out of the scope of this work. Readers are referred to [2] for an updated review of the subject. Thyroid cancer is the most common form of neoplasia of the endocrine system, accounting for about 1%–3% of all cancers, with an annual incidence in various parts of the world ranging from 0.5–10 per 100 000 [3]. The incidence of thyroid cancer is increasing, with one of the fastest rates of increase among common human cancers. Today, non-medullary thyroid cancer is the seventh most common tumor in women [4]. Improvement in cancer diagnosis can partly explain the phenomenon. However, early diagnosis alone is unlikely to account for this increase, and environmental factors must also play a role [5]. Several risk factors have been identified for thyroid carcinoma, such as radiation exposure, iodine deficiency and excess, and previous history of benign thyroid disease, such as nodules and autoimmune thyroid diseases. NMTC also has a significant gender bias, with a ratio of affected women  : men of 2.6  : 1 [6], suggesting that the hormonal environment may play an important role. The vast majority of patients with sporadic NMTC have well-differentiated tumors, papillarythyroid carcinoma (PTC), or follicular thyroid carcinoma (FTC). Papillary thyroid carcinoma accounts for approximately 80% of NMTC and is characterized by distinctive nuclear alterations including pseudoinclusions, grooves, and chromatin clearing.PTCs smaller than 1 cm are referred to as papillary microcarcinomas [7]. These tumors have been identified in up to 35% of individuals at autopsy, suggesting that they may be extremely common, although they rarely become clinically relevant. PTC can be also multifocal but is typically slow growing, with a tendency to spread to lymph nodes and usually has an excellent prognosis. Activation of the mitogen-activated protein kinase (MAPK) pathway as a result of mutually exclusive BRAF or RAS mutations or of somatic recombination of RET (RET/PTC rearrangement) or NTRK1 (TRK rearrangement) genes is found in the majority of PTCs [8, 9]. Follicular thyroid carcinoma accounts for approximately 15% of NMTC and is defined by its invasive features that result in infiltration of blood vessels and/or full penetration of the tumor capsule, in the absence of the nuclear alterations of papillary carcinoma. FTC is rarely multifocal does not usually metastasize to the regional lymph nodes but tends to spread via the bloodstream to the lung and bones. An important histologic variant of FTC is the oncocytic (Hürthle cell, oxyphilic) follicular carcinoma composed of eosinophilic cells repleted with mitochondria. Activating mutations of RAS genes, activation of the PTEN/AKT pathway (due to activating mutations of PIK3CA or loss-of-function mutations of PTEN) [10, 11], or rearrangements of the genes PAX8 and PPAR *γ*on chromosome 2q24 are often found in sporadic FTC [12]. The least common form of NMTC, accounting for less than 5% of cases, is poorly differentiated and anaplastic (undifferentiated) thyroid carcinomas, the latter representing one of the most lethal forms of human tumors. Mutations in the tumor suppressor gene p53 are typically identified in this type of carcinoma [13].

## 2. Familial Syndromes

Several syndromes are associated with non-medullary thyroid cancer such as Gardner's syndrome (familial adenomatous polyposis, FAP), Cowden disease (multiple hamartoma), Carney complex, and Werner syndrome. A summary of the clinical features and genes involved (when known) in familial syndromes associated with thyroid cancer is reported in Table 1 [14]. Gardner's syndrome or FAP results from loss-of-function mutations in the APC (adenomatous polyposis coli) tumor-suppressor gene on chromosome 5q21 and is associated with gastrointestinal adenomatous polyps, osteomas, epidermoid cysts, congenital hypertrophy of the retinal epithelium, desmoid tumors, and thyroid cancer. FAP is associated with a “cribiform-morular variant” of PTC, so called because of its unique histopathologic features. However, fewer than 2% of patients with FAP develop PTC, suggesting that PTC development is probably related to increased susceptibility rather than a specific mutation. Cowden disease, also known as “multiple hamartoma syndrome,” is associated with an increased risk of developing both benign and malignant breast tumors, and well-differentiated thyroid tumors. Eighty percent of patients have germline mutations of the PTEN (phosphatase and tensin homologue) tumor-suppressor gene. Carney complex involves both endocrine and nonendocrine organs and can affect pituitary, thyroid, gonadal, and adrenal glands. Both PTC and FTC have been associated with Carney complex type 1. The adrenal gland pathology includes a primary pigmented micronodular hyperplasia, which is the only known familial cause of adrenal Cushing's syndrome, and additional pathologies include myxomas, nevi, and schwannomas. Carney complex type 1 is associated with a mutation in the PPRKAR1*α*  (protein kinase A regulatory subunit type 1-alpha) gene. Werner syndrome is a disease of connective tissue characterized by premature aging and is often referred to as “progeria.” Patients have a predisposition to cancer, notably soft tissue sarcomas, osteosarcoma, and thyroid cancer (FTC and also PTC) which may develop as the result of genomic instability. Mutations in the RecQ helicase WRN gene have been identified, but the exact role of WRN is not known. It is possible that RecQ helicase acts as a tumor suppressor and may be responsible for genome stability.

## 3. Familial Nonmedullary Thyroid Carcinoma (FNMTC): Risk Factors and Genetic Predisposition

Patients with familial NMTC may have more aggressive tumors with increased rates of extrathyroid extension, lymph node metastases, and larger tumors in younger patients [4], underscoring the importance of accurate diagnosis. Establishing the diagnosis of a familial thyroid carcinoma may provide the opportunity for its early identification and possible prevention in family members. About 5% of patients who have well-differentiated thyroid carcinoma show a familial occurrence. The clinico-pathological features included in the analysis on familial NMTC are as reported in [15]; the mean tumor size in familial PTC/FTC is ~1.7 cm diameter. When the primary cancer site is considered in epidemiological studies, the thyroid gland shows the highest estimate of familial relative risk among all organs (between 5 and 10.4 folds as compared to ~1.8 and 2.7 for breast and colon cancer, resp., [16]). This indicates the presence of a strongly inherited component of the disease [6, 16, 17]. As for all diseases, the identification of a relevant familial recurrence risk in NMTC is complicated by the argument that families share the same environment as well as a common genetic background and it is therefore difficult to distinguish between environmental and genetic contributing factors. Although the initial familial aggregations reported over a period of time were seen as chance occurrences due to a shared environment, familial occurrence has more recently been recognized as a significant risk factor in NMTC [18]. Since the first observation of a familial case of PTC by Robinson and Orr [19] who reported PTC in twins, a number of pedigrees have been identified. It has been estimated that a family with three affected members with clinical NMTC due to environmental causes would occur by chance less than once in every 100 years. Therefore, the existence of pedigree collections of the size presented by Lesueur et al. [20] cannot occur by chance, but rather are associated with inherited predisposition. Similarly, Malchoff et al., who presented a large pedigree with PTC with 5 clinically significant cases, noted that the chance of 5 PTC occurring in a family of 23 individuals by chance would be 1 in two billion [21]. A population-based study in Norway of 5673 patients who had a first-degree relative with an index thyroid cancer, confirmed that these patients had an increased risk of developing thyroid cancer [22]. The incidence of PTC, primary pigmented nodular adrenocortical disease, and its associated conditions increased sixfold in men and fourfold in women if a first-degree relative had NMTC. Patients who have two first degree relatives with well-differentiated thyroid cancer and do not have a familial syndrome are currently classified as having familial NMTC [23]. Those who have more than two direct relatives with FNMTC have a decreased survival compared with patients who have one or two affected direct relatives [14]. In order to differentiate between FNMTC and familial syndromes associated with thyroid cancer, it is important to detect extra-thyroidal disease in patients affected with a familial syndrome. Patients who have familial NMTC have more aggressive tumors with increased rates of extra-thyroid extension, lymph node metastases, and larger tumors in younger patients. A recent work by Capezzone et al. compared the features of patients with sporadic and familial NMTC patients and tested whether FNMTC patients with parent-child relationship exhibit an “anticipation” phenomenon (earlier age at disease onset and increased severity in successive generations) [15]. In fact, significant differences between sporadic PTC and FNMTC patients included more frequent tumor multifocality (*P* = .001) and worse final outcome in FNMTC patients (*P* = .001). In addition, among FNMTC cases with parent-child relationships, an earlier age at disease presentation (*P* < .0001), diagnosis (*P* < .0001), and disease onset (*P* = .04) were found in the second generation when compared with the first generation. Patients in the second generation had a higher rate of lymph node metastases at surgery and worse outcome when compared with the first generation.

## 4. Familial Risk in FNMTC

Considering these findings, NMTC does appear to be an inherited trait. The prevalence of FNMTC has been estimated to be between 2.5% and 8% of the total NMTC patients. The majority of FNMTC pedigrees are small [20]; the pattern of inheritance is usually autosomal dominant, but since a large number of pedigrees present a significant lack of penetrance, polygenic inheritance cannot be ruled out [20, 24]. The variable expression of FNMTC suggests that the responsible gene(s) may lead to predisposition or susceptibility to thyroid cancer. In addition to NMTC, members of FNMTC pedigrees may present a spectrum of benign thyroid diseases including follicular adenoma, diffuse and multinodular goitre, and autoimmume thyroiditis. As mentioned above, these diseases are considerably more common in the general population than NMTC. However, the high occurrence of these diseases in FNMTC pedigrees suggests that they are a part of FNMTC, and pedigree members are therefore considered to be at increased risk of benign thyroid disease [18, 23].

## 5. FNMTC Susceptibility Loci

In order to identify the gene(s) responsible for thyroid cancer predisposition, several groups have undertaken genome wide linkage analysis using microsatellite markers evenly distributed across the genome and informative large pedigrees with multiple affected members. To date, four different loci have been identified for genetic predisposition to familial NMTC. The first locus to be implicated in familial PTC was MNG1, which maps to chromosome 14q31. In this case, the authors studied a family from Montreal, Canada, with 18 affected with multinodular goitre (MNG), two of whom presented with PTC and one with follicular adenoma. The predisposing locus was identified by linkage analysis with a maximal LOD (Logarithm Of Odds, a genetic measure of the significance of linkage) score of 4.88 distal to marker D14S1030, the region of interest spanning around 1 Mb [25]. The locus was confirmed in other families with MNG [26], but no evidence of linkage was found in additional FNMTC pedigrees suggesting that only a small percentage of FNMTC can be attributed to this locus [20, 27]. A second locus, PRN1, was identified by Malchoff et al. on chromosome 1q21 in an American family with five members affected by PTC, one by colon cancer and two by papillary renal carcinoma [28]. The susceptibility locus was identified at 1q21 with a maximum LOD score of 3.58 in a critical region of 20 cM This region encompasses the NTRK1 gene, but no mutation segregating with the disease was found among FNMTC pedigrees [27]. To date, linkage to this locus has not been confirmed in any other independent study. The existence of a susceptibility locus for familial NMTC (NMTC1) on chromosome 2q21 was first identified in a large Tasmanian pedigree with recurrence of papillary thyroid carcinoma (PTC) [29]. In the same study, linkage analysis of 80 FNMTC pedigrees gave a significant LOD score of 3.07 at marker D2S2271. Stratification based on the presence of at least one case of the follicular variant of PTC, the phenotype observed in the Tasmanian family increased the corresponding LOD score for chromosome 2, suggesting that this locus may be strictly correlated to the follicular variant of PTC. Finally, the fourth susceptibility locus is TCO (Thyroid Carcinoma with Cell Oxyphilia) mapped on chromosome 19p13.2 by Canzian et al. [30]. The study analysed a large French pedigree with six patients affected by MNG and three by PTC. A review of the histology indicated that all cases examined presented oncocytic features. Oncocytic tumors, also known as oxyphilic tumors and in the thyroid gland as Hürthle cell tumors, are made of cells replete with mitochondria with a “swollen” (i.e., “oncocytic,” from the Greek word onkoustai, to swell) appearance. Oncocytic tumors may occur at various sites (see [31], for a review) but they are more frequently observed in the thyroid gland. Thyroid oncocytic tumors originate from follicular cells, with the exception of the rare oncocytic variant of medullary carcinoma. They can be benign (oncocytic adenomas) or malignant (oncocytic carcinomas). Oncocytic tumors in the thyroid are regarded as special subtypes, since their features are distinct enough to set them apart from corresponding neoplasms lacking mitochondrial accumulation. Accordingly, oncocytic thyroid carcinomas are classified as variants of FTC (more commonly) or of PTC (less commonly) [1]. Using this distinctive phenotype for data analysis led to the successful identification of a predisposing locus for thyroid cancer with oncocytic features on chromosome 19p13.2, with a significant LOD score at marker D19S916 (3.01). The TCO locus extends up to ~2 Mb and has been further refined to a 1.6 Mb interval by adding more markers and more families with affected individuals showing oncocytic tumors [32]. It is worth noting that linkage to the TCO locus has been subsequently confirmed in independent studies [18, 27]. Furthermore, analysis of additional families has provided evidence for the genetic interaction between the TCO at 19p13 and NMTC1 at 2q21 loci, resulting in a significantly increased risk of NMTC in patients that carry both susceptibility loci [32]. Several groups have undertaken a positional candidate gene approach to identify the causative mutation underlying TCO predisposition, taking into account the specific phenotype of thyroid oncocytic tumors, that is, the abnormal proliferation of mitochondria and the presence of a severe bioenergetic defect [33–37]. Our group recently defined the bioenergetic defect in vitro in the only existing thyroid oncocytic tumor cell model, the XTC.UC1 cell line. Two mutations were identified in mitochondrial DNA (mtDNA) which affect complex I and III of the OXPHOS respiratory chain [38]. One of the mutations is a single base pair insertion in the ND1 complex I gene, causing a premature stop codon and the absence of the protein as evaluated by western blot analysis. The second is a missense mutation in the CYTB complex III gene. These two mutations markedly decrease the activity of both respiratory complexes and explain the defective ATP synthesis previously reported [33, 34]. Subsequently, the high occurrence of pathogenic mutations impairing complex I has been confirmed by our group in vivo in sporadic thyroid oncocytic tumors, leading to the conclusion that disruptive complex I mutations may well be considered to be the hallmarks of the oncocytic phenotype [39]. The close association between disruptive complex I mutations and the oncocytic phenotype has also been confirmed in other types of oncocytic tumors [40–43]. Since the human complex I is composed of 45 subunits, only 7 of which are mtDNA-encoded, our search for the defective nuclear gene on chromosome 19p13.2 began with the analysis of genes encoding mitochondrial complex I subunits and mapping to the TCO locus. So far, somatic and germline missense mutations have been reported in the *NDUFA13 *gene (previously known as GRIM19) [44], a member of complex I and a key regulator of cell death. Germline mutations have been reported by our group in *TIMM44*, a component of the mitochondrial translocon complex for importing nuclear-encoded proteins into the mitochondria [45]. However, the effect of these mutations in TCO predisposition is still uncertain, since either no functional studies have been presented (at least for *NDUFA13*) or they did not reveal a clear negative effect in functional molecular studies [45]. Moreover, the family in which the locus was originally mapped [30] did not carry any coding mutation in *NDUFA13 *and *TIMM44*, suggesting either that these genes are not directly involved in the TCO etiology or at least the presence of genetic heterogeneity in TCO. Several other genes mapping to the region encode proteins involved in mitochondrial functions and/or with a known role in tumor development, and these have been subsequently screened. A list of all the genes so far analysed without identifying etiological variants is reported in Table 2 (our unpublished results). Thus, the search for the TCO predisposing variants is still open. The application of next-generation sequencing technologies capable of sequencing of ~2 megabases of genomic DNA in a single experiment, will allow the entire TCO region to be resequenced, and not just the coding region of genes, a limit of the traditional candidate gene approach. Identification of the relevant mutation(s) will surely benefit the characterization of TCO.

## 6. Association Studies in Thyroid Cancer

FNMTC has been identified as a clinically distinct entity from its sporadic counterpart, and although the mutated genes(s) have not yet been identified, the genetic basis underlying its predisposition has been recognized by the scientific community. However, several studies have recently reported the identification of predisposing common variants in association-based case-control studies using single nucleotide polymorphism (SNP) high throughput genotyping [46–49]. One of the most significant studies was a genome-wide association scan in a large cohort of Icelandic cases with thyroid cancer and respective controls, followed by a replication study in individuals of European descent [49]. The authors demonstrated that two common variants, located on 9q22.33 and 14q13.3, are associated with the disease. Overall, the strongest association signals were observed for rs965513 on 9q22.33 (OR = 1.75; *P* = 1.7 × 10^−27^) and rs944289 on 14q13.3 (OR = 1.37; *P* = 2.0 × 10^−9^). The gene closest to the 9q22.33 locus is *FOXE1 *(TTF2), and *NKX2-1 *(TTF1) is among the genes located at the 14q13.3 locus. Both variants contributed to an increased risk of both papillary and follicular thyroid cancer. Approximately 3.7% of individuals were homozygous for both variants, and their estimated risk of thyroid cancer was 5.7-fold greater than that of noncarriers. In a study using a large sample set from the general population, both risk alleles are associated with low concentrations of thyroid stimulating hormone (TSH), and the 9q22.33 allele was associated with low concentration of thyroxin (T(4)) and high concentration of triiodothyronine (T(3)). Another interesting study, although not specific for thyroid cancer as such, in an isolated population of Sardinians identified a strong association (*P* = 1.3 × 10^−11^) between rs4704397 and circulating TSH levels [48]. Serum TSH is a sensitive indicator of thyroid function, and overt abnormalities in thyroid function lead to common endocrine disorders. Each additional copy of the minor A allele was associated with an increase in TSH. The marker is located in intron 1 of *PDE8B*, encoding a high-affinity cAMP-specific phosphodiesterase. The association was replicated in genetically distant cohorts from Tuscany and Old Order Amish (overall *P* value = 1.9 × 10^−20^). It will be very interesting to analyse the association of the above-mentioned SNPs with the familial form of non-medullary thyroid carcinoma.

## 7. Conclusions

Familial NMTC represents 3%–7% of all thyroid tumors and is associated with some of the highest familial risks among all cancers, with a risk of developing this type of neoplasia for first-degree relatives of 5–10-fold compared to the general population. Several predisposing loci have been identified in the recent years but the mutated gene has not been identified yet. However, the new technologies already available have rendered the resequencing of large genomic regions from defined linkage intervals reasonably possible in single experiments, and the application of these methods will greatly speed up the search for the NMTC gene(s). It will then become possible for NMTC families to receive careful genetic counseling for all at-risk members similarly to what is already happening for FMTC. In addition, the study of FNMTC genetics, and in particular familial TCO represents a key model for the study of nuclear and mitochondrial crosstalk in thyroid tumor development. The thyroid gland is characterized by a high energetic metabolism and any genetic defect (either of nuclear or mitochondrial origin) leading to bioenergetic deficits may lead to an alteration of this crosstalk and to an alteration of the mitochondrial phenotype, as seen in TCO. Identifying the causative mutations involved in these energetic defects will also provide a powerful tool to design new and specific treatments for such tumors.

## Figures and Tables

**Table 1:** Familial syndromes with associated non-medullary thyroid cancer.

Syndrome	Clinical Features	Inheritance pattern	Locus	Gene
Gardner's syndrome (FAP)	gastrointestinal adenomatous polyps, osteomas, epidermoid cysts, hypertrophy of the retinal epithelium, desmoid tumors, PTC (cribiform-morular variant)	Autosomal dominant	5q21	APC

Cowden disease	hamartoma, breast cancer, PTC, FTC	Autosomal dominant	10q22	PTEN

Carney complex	pituitary, gonadal, and adrenal gland cancer, PTC, FTC	Autosomal dominant	17q23-24	PRKAR1a

Werner syndrome	Premature aging, soft tissue sarcomas, osteosarcoma, FTC/PTC	Autosomal recessive	8p11-p12	WRN

**Table 2:** New changes in cDNA of TCO candidate genes on 19p13.2.

PCR product	Change in cDNA	Type of aa change	Het frequency in TCO patients (%)	Het frequency in controls (%)
*TIMM44*x4	G344A C925A	silent P308Q	12.5 12.5	na 0 0 na na
*TIMM44*x9	G1274A G509A	silent silent	12.5 12.5	na na 22.5 3.0
*TIMM44*x13	T971C G868T	silent 3′UTR	12.5 12.5 8.0	na na na na
*ELAVL1*x3	C591A G1111C	silent R219P	42.9 13.0 25.0 14.2	na na na na
*ELAVL1*x5	Δ(CTTCCACTTCA	3′UTR 5′UTR	12.5 12.5	na na na na
*CCL25*x5	AC) bp1267-1280	T289M	37.5 50.0 37.5 50.0	na na 7.3 na na
*LASS1*x3	C182T A866T	K1266N	37.5 37.5	
*MARCH2*x5	A3798C C4996T	silent Y1897D	37.5 12.5 37.5 37.5	
*MARCH2*x5	T5692G G9569C	S3189T	12.5 50.0	
*GRIM19*x1	A21814G T21858A	I7271V silent	12.5	
*EDG5*x2	G26790A C25572T	silent silent		
*MUC16x1 *	T25638G A27791C	D8546E		
*MUC16x1 *	C31905T G31721A	N9263T		
*MUC16x1 *	G36955A G39400A	silent R10573H		
*MUC16x2 *	C41705T	E12318L		
*MUC16x3 *		V13133M		
*MUC163x *		P13901L		
*MUC16x3 *				
*MUC16x3 *				
*MUC16x3 *				
*MUC16x3 *				
*MUC16x5 *				
*MUC16x5 *				
*MUC16*x15				
*MUC16x39 *				
*MUC16x63 *				

na= not available. The changes cosegregating are reported. Only for TIMM44 missense change, the variant was not found in a large group of controls. The other genes screened so far but with no new changes identified are *NDUFA7, PPAN, FBLX12, ICAM1, ICAM4, LASS1, LASS4*, *SMARCA4, DNM2*, and *ANGPL4; RAB11B*; *ADAMTS10*; *PIN1*; *UBL5;* and* KEAP1*.
